# A mathematical model of COVID-19 transmission in a tertiary hospital and assessment of the effects of different intervention strategies

**DOI:** 10.1371/journal.pone.0241169

**Published:** 2020-10-26

**Authors:** Yae Jee Baek, Taeyong Lee, Yunsuk Cho, Jong Hoon Hyun, Moo Hyun Kim, Yujin Sohn, Jung Ho Kim, Jin Young Ahn, Su Jin Jeong, Nam Su Ku, Joon-Sup Yeom, Jeehyun Lee, Jun Yong Choi

**Affiliations:** 1 Department of Internal Medicine, Severance Hospital, Yonsei University College of Medicine, Seoul, Republic of Korea; 2 Department of Mathematics, Yonsei University, Seoul, Republic of Korea; 3 Department of Computational Science and Engineering, Yonsei University, Seoul, Republic of Korea; Azienda Ospedaliero Universitaria Careggi, ITALY

## Abstract

Novel coronavirus (named SARS-CoV-2) can spread widely in confined settings including hospitals, cruise ships, prisons, and places of worship. In particular, a healthcare-associated outbreak could become the epicenter of coronavirus disease (COVID-19). This study aimed to evaluate the effects of different intervention strategies on the hospital outbreak within a tertiary hospital. A mathematical model was developed for the COVID-19 transmission within a 2500-bed tertiary hospital of South Korea. The SEIR (susceptible-exposed-infectious-recovered) model with a compartment of doctor, nurse, patient, and caregiver was constructed. The effects of different intervention strategies such as front door screening, quarantine unit for newly admitted patients, early testing of suspected infected people, and personal protective equipment for both medical staff and visitors were evaluated. The model suggested that the early testing (within eight hours) of infected cases and monitoring the quarantine ward for newly hospitalized patients are effective measures for decreasing the incidence of COVID-19 within a hospital (81.3% and 70% decrease of number of incident cases, respectively, during 60 days). Front door screening for detecting suspected cases had only 42% effectiveness. Screening for prohibiting the admission of COVID-19 patients was more effective than the measures for patients before emergency room or outpatient clinic. This model suggests that under the assumed conditions, some effective measures have a great influence on the incidence of COVID-19 within a hospital. The implementation of the preventive measures could reduce the size of a hospital outbreak.

## Introduction

In late December 2019, an outbreak of an emerging disease (COVID-19) due to a novel coronavirus named severe acute respiratory syndrome coronavirus 2 (SARS-CoV-2) originated in Wuhan, China and rapidly spread across China and beyond. This outbreak began from a seafood and live animals whole-sale market in Wuhan, but cases of patients suffering from the infection have been documented both in hospital and in family settings [1]. People become infected by respiratory droplets from coughing and talking but aerosol transmission is also possible in cases of protracted exposure to elevated aerosol concentrations in closed spaces [2]. Transmission may occur indirectly through touching a contaminated surface, followed by touching their eyes, nose, or mouth. The coronavirus may also be unexpectedly transmitted by an asymptomatic carrier [3]. In fact, patients considered asymptomatic released large amounts of viruses at the early phase of the infection, which posed enormous challenges to contain the spread of COVID‐19 [4], but 97.5% of patients with COVID-19 developed symptoms within 11.5 days [5].

Novel coronavirus can spread widely in confined settings, including hospitals, cruise ships, prisons, and places of worship [6]. In particular, a healthcare-associated outbreak could become the epicenter of COVID-19. Transmission in a hospital raises serious problems since many immunocompromised and aged patients live together and an outbreak in a hospital could paralyze its role of providing essential medical care within the healthcare system. Therefore, effective strategies to contain COVID-19 outbreaks in hospitals are required [7]. However, even for a well-established hospital, coping with the unprecedented COVID-19 outbreak would be a complex challenge.

In this study, we developed a mathematical compartment model to predict COVID-19 transmissions over time in a tertiary hospital, and to evaluate the effectiveness of different intervention strategies.

## Methods

### Mathematical model

We divided individuals into four infection classes: susceptible, exposed, infectious, and removed. (i.e., recovered, or otherwise no longer infectious). The susceptible, *S*, represents the people who can be infected by SARS-CoV-2. The exposed, *E*, are those already infected but who did not recognize the disease and even front door screening could not detect it. The infectious, *I*, and removed, *R*, follow the usual immunizing infection [8].

**Fig 1:**
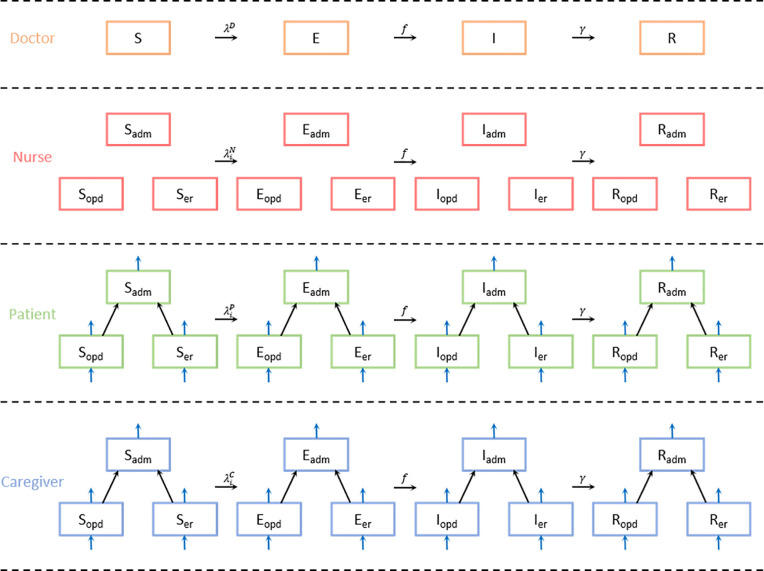
The diagram for the SEIR (susceptible-exposed-infectious-recovered) model with compartments of doctor, nurse, patient, and caregiver. We assumed doctors work across departments while nurses work in their own departments; therefore, individuals in the hospital are divided into 10 statuses. Blue arrows refer to the in- and out-flow of patients and caregivers in the OPD and ER, with only inbound arrows for those in ADM. Abbreviations:—adm: admission; opd: outpatient department; er: emergency room.

The study hospital consists of three main categories: ward, outpatient clinic, and emergency room. We divided people who entered the hospital into four main compartments: Doctor, Nurse, Patient, and Caregiver. Doctors and nurses are a part of the medical staff who are fixed in the hospital, while patients and caregivers are visitors who vary from day to day. We assumed doctors work across departments while nurses work in their own departments. Therefore, individuals in the hospital were divided into 10 statuses: doctors as a whole and nurses, patients, and caregivers in each department (Fig 1).

Because there are three factors that divided the population, we represented the compartment in the form of $X_{Z}^{Y}$. *X* indicates 4 infection classes. *Y* is the occupation and *Z* represents the department to which the component belongs. We used the notations *D* for the doctors’ group, *N* for the nurses’ group, *P* for patients’, and *C* for caregivers’. According to convention, we denoted ADM as the ward, OPD for the outpatient department, and ER for the emergency room. The component of WAIFW (Who-Acquires-Infection-From-Whom) matrix $W_{\left(i,i_{k})(j,j_{k}\right)}$ represents the transmissibility from the (*j*,*j*_*k*_)-th infectious group to the (*i*,*i*_*k*_)-th susceptible group, where the index (*i*,*i*_*k*_) indicates *i*-th occupation and *i*_*k*_-th department. Note that the compartment for the doctors’ group does not have the subscript *Z*.

Y={D=1,N=2,P=3,C=4}

Z={ADM=1,OPD=2,ER=3}

Usually, the exposed person does not participate in the infection, but this is not clear in case of COVID-19. Therefore, we assumed the exposed person is involved in the FOI (Force of Infection) *λ* with transmissibility reduced by *ε*. In this research, we set this value as 0.1.

$$\lambda_{i_{k}}^{i}=\sum_{j\in Occupation}\sum_{j_{k}\in Depeartment}W_{\left(i,i_{k})(j,j_{k}\right)}(\varepsilon E_{j_{k}}^{j}+I_{j_{k}}^{j})$$

Public health authorities define a significant exposure to COVID-19 as face-to-face contact with a symptomatic patient within six feet that is sustained for at least a few minutes. We estimate the contact rate matrix, *C*, based on the short survey and employ the reproductive number, *R*_0_, from the literature. Setting the population vector *η* as the number of staff, and the stabilized number of inflow and outflow to each department for visitors, we construct the WAIFW matrix, *W*, by assuming that it is proportional to the contact rate matrix [8, 9]:

W=qC

The proportionality factor q represents the transmission risk per contact, which can be calculated through the relation between WAIFW and R_0_.

$$q=\frac{R_{0}}{\rho (C\circ \eta )(\varepsilon /f+1/\gamma )}$$

Here *f* is the rate that at which the exposed becomes infectious, and the *γ* is the rate that the infectious would recover. The population vector η denotes the number of staff, and the stabilized number of inflow and outflow to each department for visitors and ρ(C∘η) is the spectral radius of the resulting matrix multiplying each row of C by corresponding element of η.

Fig 1 shows the diagram for the SEIR (susceptible-exposed-infectious-recovered) model with compartments of doctor, nurse, patient, and caregiver. The patients and caregivers in OPD and ER come in and out, but ADM does not have the inbound arrows, as we assumed that patients are not directly admitted to the ward from the outside but only from other departments.

**Fig 2:**
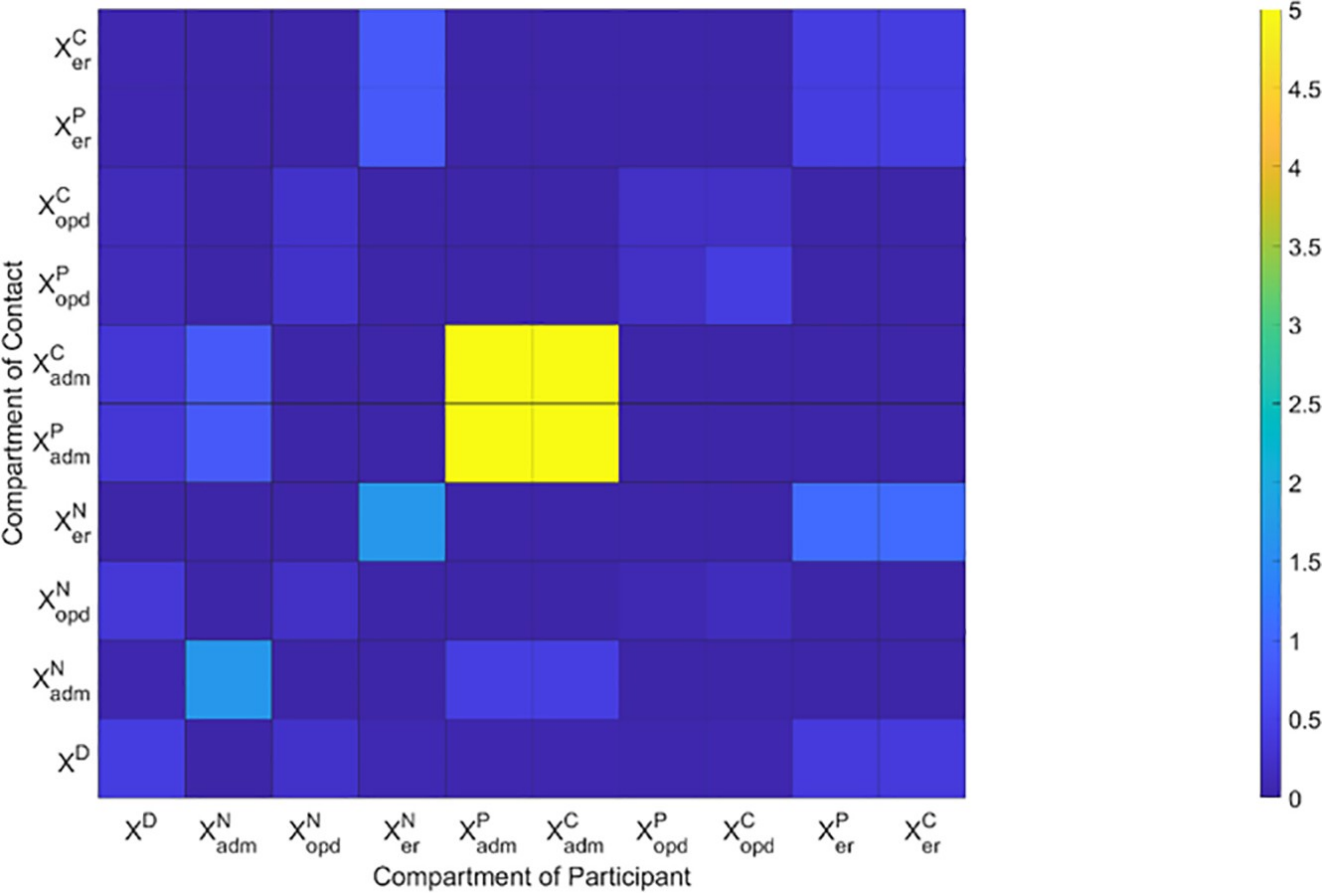
Average contact duration matrix indicating the average hours of contact in a day among medical staff and visitors. The horizontal axis denotes the compartments with the contacts and the vertical one denotes the compartments that are contacted. The matrix meets the reciprocity of contacts which makes the contact rate matrix symmetric.

Average contact duration matrix indicates the average hours of contact in a day among medical staff and visitors (Fig 2). The horizontal axis indicates the compartments having the contacts and the vertical one indicates the compartments that are contacted. The matrix meets the reciprocity of contacts which makes the contact rate matrix symmetric.

### Study setting and ethics statement

The study site was Severance hospital, a tertiary care hospital with 2500 beds in South Korea. There are 74 wards for inpatients within the study hospital, and we assumed each unit had the same capacity of doctors and nurses. If two doctors work in a unit, there are 148 doctors working in wards daily. Other numbers based on epidemiology are shown in Table 1.

**Table 1:** Number of hospital staff.

Department	Occupation	Number (persons)
**OPD**	Doctors	194
	Nurses	1300
**Ward (74 units)**	Doctors	148
	Nurses	500
**ER**	Doctors	18
	Nurses	50
**TOTAL**	Doctors	360
	Nurses	1850

Data are presented as number based on epidemiology of the study site.

Abbreviations: OPD: outpatient department; ER: emergency room.

We retrieved data from the hospital administration department on the number of patients who were admitted from the ER, hospitalized in wards, and had gone to outpatient clinics from February 24, 2020 to March 13, 2020. The hospital administration department provided us with the data without identifiable private information.

Before we began this study, we confirmed with the Institutional Review Board (IRB) of Severance Hospital that ethics approval was not needed, since we did not utilize any personal or identifiable information of the patients. Authors who were affiliated to the hospital devised parameters through anonymized data and work experience in the study site.

### Parameters

At outpatient clinics, some patients are accompanied by caregivers, and we assumed half of outpatients visit clinics with one caregiver. On the other hand, we assumed one caregiver was assigned per patient in each ward and the emergency center as a hospital policy.

We arbitrarily assumed 10 exposed and 10 infectious people from the patient and caregiver group came into the ER every three days, while the same number from the patient group and half of them from the caregiver group came into the OPD every five days. We presumed they had not been diagnosed by any reason when they entered the study hospital, which meant they were not detectable with the known data.

All other parameters for the model are shown in Table 2.

**Table 2:** The base parameter settings.

Parameter	Symbol	Value
Incubation Period [days]*	1/*f*	5.2
Infectious Period [days]*	1/*γ*	9.5
Impact of the exposed onto the infection^×^	*ε*	0.1
The average inflow number of ADM from the outside per day†	-	0
The average inflow number of OPD from the outside per day†	-	11242.6
The average inflow number of ER from the outside per day†	-	209.3
The average number from ER to ADM per day†	-	51.4
The average number from OPD to ADM per day†	-	314.6
The rate of outflow from the ADM [1/days]†	-	0.1491
The rate of outflow from the OPD [1/days]†	-	6
The rate of outflow from the ER [1/days]†	-	4

* The incubation period and infectious period are from reference [13], [17]

× Rate at which the exposed persons become infectious

^†^ An average of data collected from the hospital administration department in the study site

Abbreviation:—ADM: admission; OPD: outpatient department; ER: emergency room.

### Intervention scenarios

**Fig 3:**
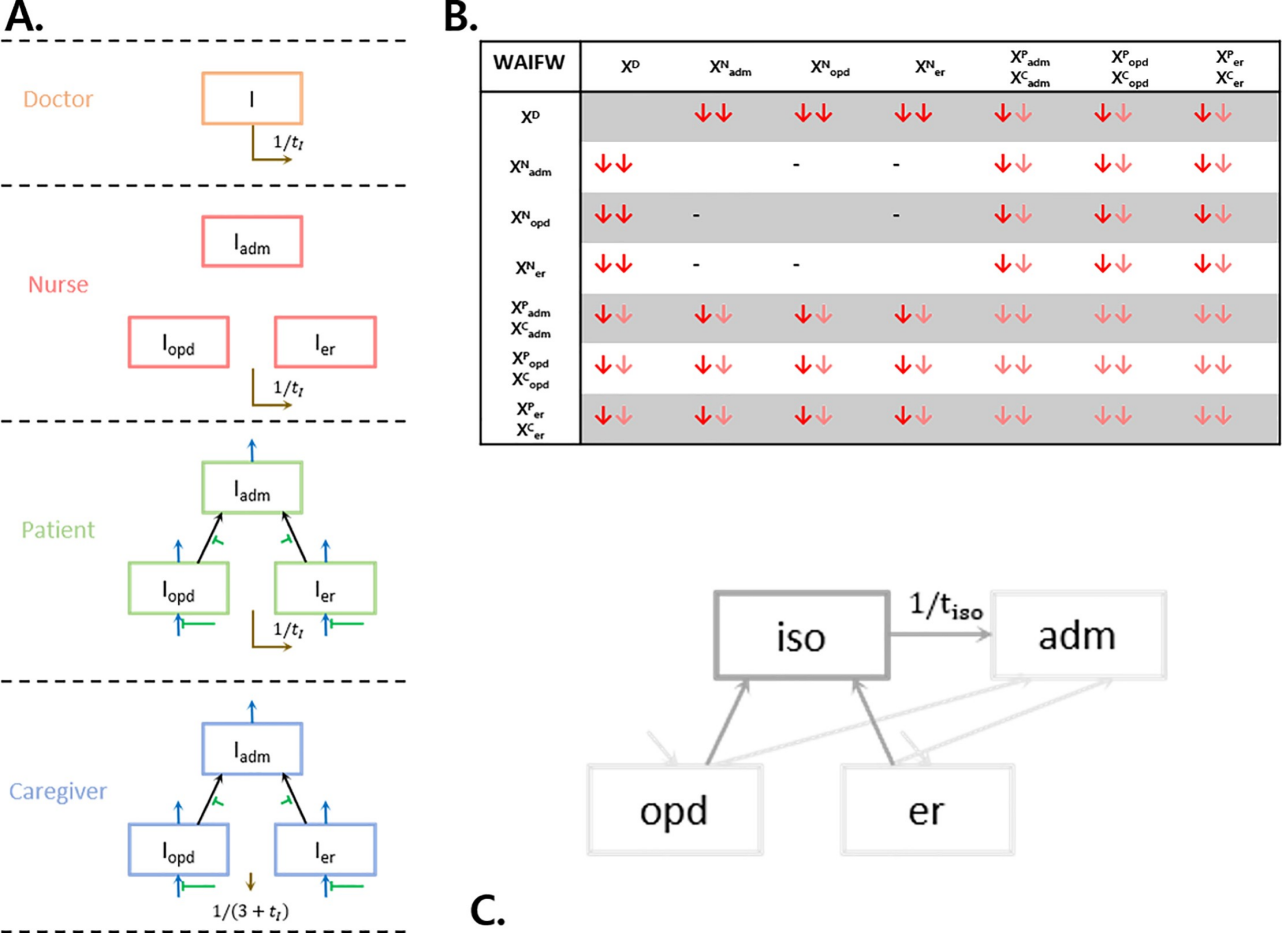
Intervention scenario. (A), Infectious class in SEIR diagram; green arrows indicate front door screening and brown arrows indicate testing the infectious patients. Front door screening intervenes the inflow of the infectious patients and the tested patients are removed at the rate of 1/t. As we described, we assumed caregivers are tested three days later than other groups. (B), WAIFW diagram of each group; pink arrows indicate reduction of transmission by wearing a universal mask while red arrows are reinforcement of the protection device among medical staff. Reinforcing the protection device among medical staff reduces the probability of transmission as in red arrows. (C), Diagram for pre-isolating the patients who are to be admitted, which we named the quarantine unit. Patients are usually admitted from OPD and ER (light gray arrows), but if all patients are directed to the isolated ward (iso) (dark gray arrow), isolated people are out of the dynamics for 14 days. Abbreviations:—adm: admission; opd, outpatient department; er: emergency room; iso: quarantine unit.

The study site had implemented several controlling measures to prevent outbreaks within the hospital (Table 3) but, in this mathematical model, we set up four intervention scenarios for the model (Fig 3).

**Table 3:** Control measures to prevent COVID-19 outbreak in the study site.

Measures	Description
**Front door screening**	Fever screening with thermometer and infrared camera
	Inquiring about contact history and related symptoms (cough, sputum, sore throat) by a standard checklist
**Triage clinics for high risk group**	Separate clinics for persons who have either fever or respiratory illness, and who have a history of traveling abroad or visiting high risk areas, located in front of the entrance to the outpatient clinic and emergency room.
**Access control**	The study site minimized the number of entrances to hospital buildings and restrict visiting to admitted patients
**Universal mask wearing**	All healthcare workers, employees, patients, and visitors are obligated to wear masks in the hospital
**Increasing diagnostic capability for COVID-19**	Real-time Reverse Transcription Polymerase chain reaction (RT-PCR) for any patients with related symptoms or suspected findings without specific causes The frequency of real-time RT PCR testing to diagnose COVID-19 in the study hospital has increased from 1 time/day to 6 times/day during COVID-19 outbreak since January 2020 The test results could be reported within four hours
**Pneumonia preemptive isolation ward**	Isolation ward with negative pressure isolation rooms in operation Patients with either fever or pneumonia were preemptively isolated and treated within the ward Healthcare workers for those patients are required to wear appropriate personal protective equipment (PPE).

### Front door screening

According to one study, on admission, 43.8% of COVID-19 patients presented a fever, 67.8% a cough, and 33.7% with sputum [1]. The sensitivity is the test’s ability to correctly designate a subject with the disease as positive, and we calculated the sensitivity of front door screening at 0.5. However, if we sought an epidemiologic relationship to confirmed patients or travel history along with their current symptoms, the sensitivity would increase, which we assumed at 0.7. Front door screening was performed on visitors of three different departments: AMD, OPD, and ER.

### Quarantine unit for newly admitted patients

Even though the study hospital executed a pneumonia preemptive isolation unit, asymptomatic patients (usually in the exposed group) could be missed in this control. Therefore, we assumed all patients who were admitted either from the ER or outpatient clinics were sent to a quarantine unit for two weeks.

### Early testing (within eight hours) of suspected people to detect the disease

We assumed the average time for diagnosis would be eight hours. As COVID-19 patients in the hospital were confirmed, they were directed to isolated rooms (the removed group). As medical staff were aware of the clinical symptoms of COVID-19 and patients’ medical conditions were regularly and closely monitored, the groups were immediately tested when the related symptoms occurred. However, caregivers were not as attentive as patients were; therefore, we assumed they were tested three days later than other groups.

### Personal protective equipment for both medical staff and visitors

The regulation of the study hospital specifies that all people in the hospital are required to wear masks. Since SARS-CoV-2 is a respiratory virus like other coronaviruses or influenza, facial masks significantly reduce transmission of human coronavirus from symptomatic individuals, which could be a way to control of COVID-19 [10]. However, because the chance of catching COVID-19 from a passing interaction in a public space is minimal, some people raise the question of effectiveness of universal use of masks by all healthcare workers and visitors [11]. However, the effect of face masks, respirators and eye protection that result in reducing risk of outbreak had been verified [12]. We assumed the protection rate of transmission by masks would be 0.3, and the protection rates could reach 0.3, 0.6, or 0.9 as reinforcing personal protective equipment (PPE) with gloves, gowns, eye protection, etc.

Fig 3 shows the above interventions diagrammatically.

### Sensitivity analysis

#### Reproductive number

The vulnerability of results for each intervention required checking as the reproductive number varied since we set the WAIFW matrix from it. We employed the value 2.2 from the paper for early transmission analysis of COVID-19 in Wuhan [13]. However, over time, many studies have been performed regarding an estimation of the reproductive number in different circumstances with control measures [14, 15]. Variability must be taken into consideration within the range of reproductive numbers for evaluating each intervention. Therefore, we also simulated the impact of our interventions in case of a very high reproductive number (6.47) [14].

#### Incubation period and serial interval

The incubation period has not been determined yet and we set it at 5.2 days [13] as a base case and 6.4 days [16] for sensitivity analysis. The serial interval has not been determined and we assumed 9.5 days [17] and 4.6 days for sensitivity, which is 2 times of 2.3 days–that is the difference between 7.5 days serial interval and 5.2 days incubation period [13]. Note that these parameter values were to be fitted with different assumptions for distribution. However, in an average sense, they have few differences with other fitting results and can be used as parameters in our model. With these parameters, we set the base-, worst-, and best-case scenarios and performed the sensitivity analysis with them (See Table 4).

**Table 4:** Parameter values for evaluation of various interventions and sensitivity analysis*.

Scenario	Parameter Set		Source
**Base**	1/*f*^†^	5.2	[13]
	1/*γ*^‡^	9.5	[17]
	*R*_0_^§^	2.2	[13]
**Best**	1/*f*	6.4	[16]
	1/*γ*	9.5	[17]
	*R*_0_	2.2	[13]
**Worst**	1/*f*	5.2	[13]
	1/*γ*	4.6	[18]
	*R*_0_	6.47	[14]

* We set the best- and worst-case scenario parameter sets in terms of curbing viral transmission. If the virus has a long infectious period and low reproductive number, the transmissibility is low, which is helpful in curbing the spread of disease. On the other hand, with a short infectious period and high reproductive number, it would lead to high transmissibility even in a restricted condition.

^†^ 1/*f* is the incubation period; a reversal of the rate at which the exposed patients become infectious

^‡^ 1/*γ* denotes the infectious period, a reversal of the rate at which the infectious patients would recover

^§^ *R*_0_ denotes the reproductive number, an average number of secondary cases generated by a case in an entirely susceptible population.

## Results

### The dynamics of COVID-19 transmission without any intervention

**Fig 4:**
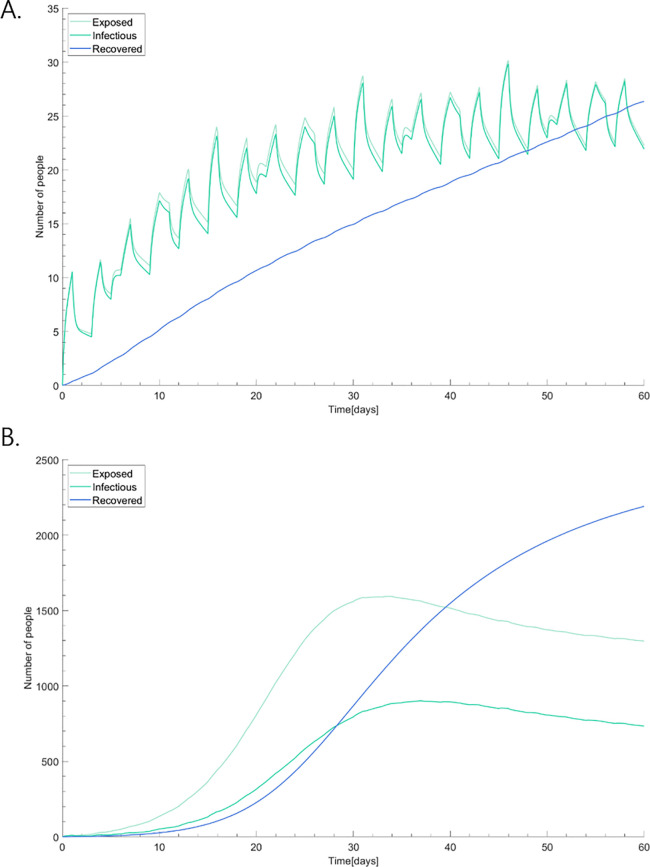
Daily new incidence of COVID-19. (A) Epidemics in doctor status. (B), Epidemics in 10 statuses; from top to bottom, ADM, OPD, ER. Abbreviations:—ADM: admission; OPD: outpatient department; ER: emergency room.

**Fig 5:**
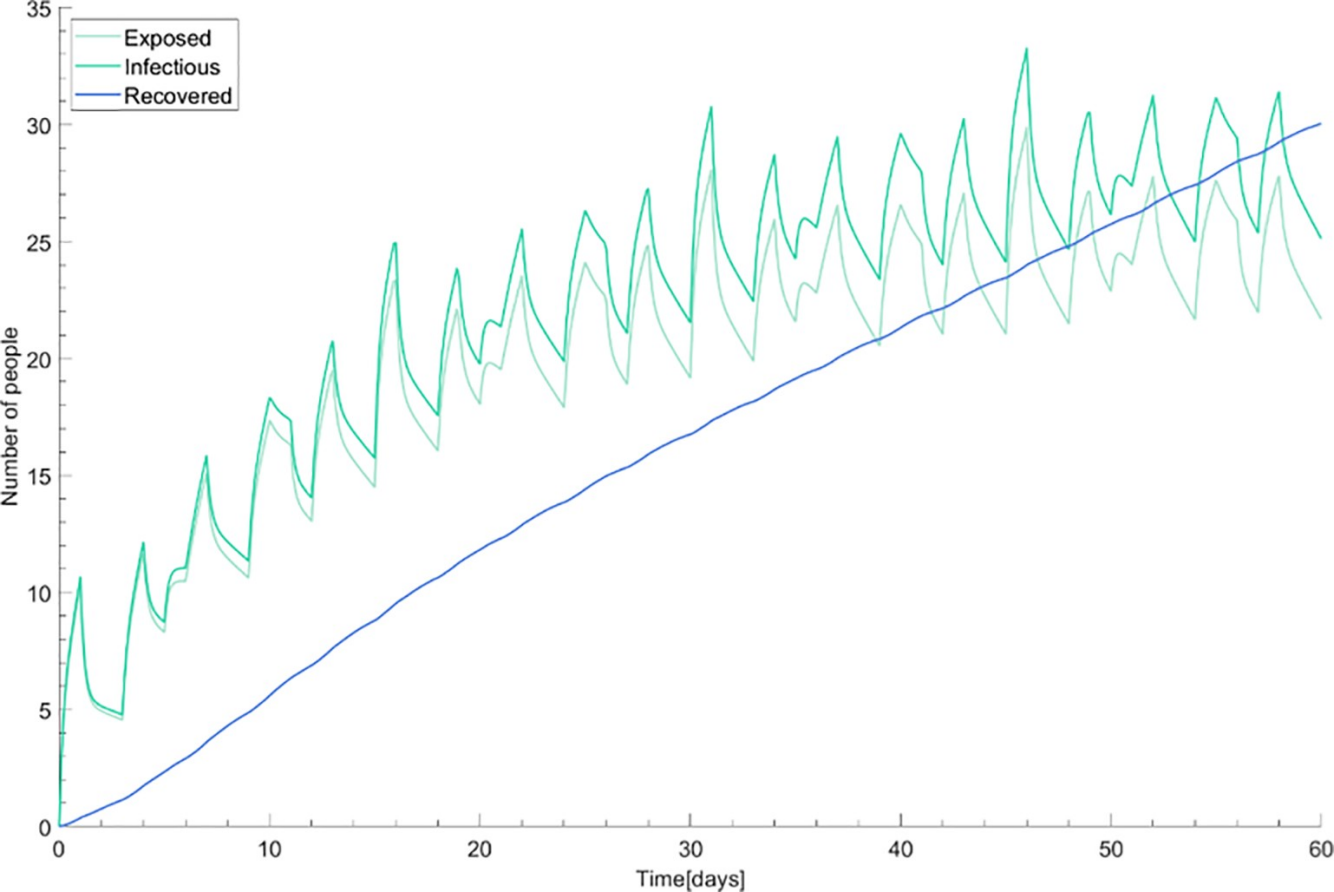
Total epidemic curve of COVID-19. The curve of the recovered patients represents a trend of totally susceptible population in the hospital before the outbreak being immune to the disease.

The model simulated the epidemic curve of COVID-19 in the study site with base parameters. Fig 4 shows the daily new incidence of COVID-19 without any intervention for 60 days. The horizontal axis represents time (days) and the vertical axis indicates the number of people who are newly confirmed patients within the past 24 hours. This predicts daily new cases of infected people from four compartments over time within the hospital. Since doctors work across departments, their epidemic is shown in Fig 4A. Other groups work or stay in separate departments, which indicate different epidemics (Fig 4B). The infected cases in ER and OPD are very small due to relative short duration of stay and low reproductive number, so the spread is restricted and there is no remarkable outbreak in the ER or OPD. However, when confirmed cases are in wards, they become more transmissible since visitors have a high contact duration matrix within the group. Fig 5 indicates total epidemic curves of COVID-19. The number of the exposed and infectious people among visitors grows in the early period but this reaches a plateau. The curve of the recovered patients represents a trend of a totally susceptible population in the hospital before the ourbreak being immune to the disease. The total number of infectious people is about 30 at the end of the dynamics.

### The effects of various interventions

**Fig 6:**
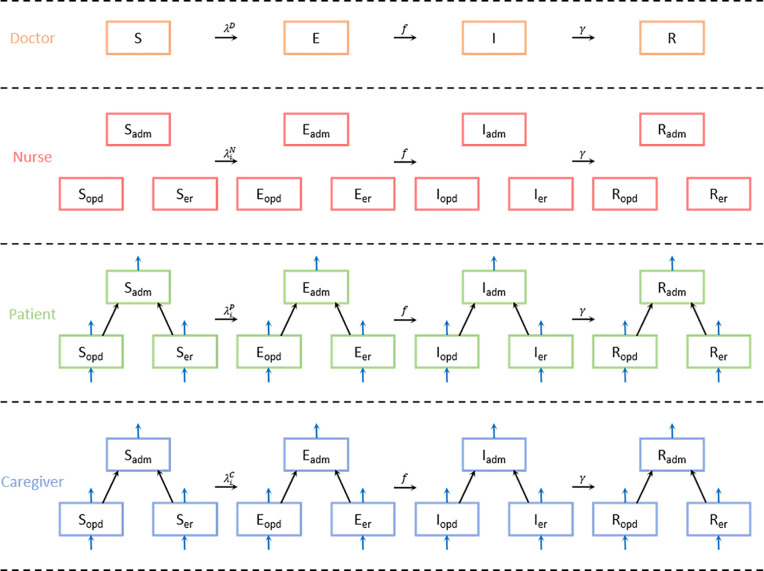
Effectiveness of all intervention scenarios. Effectiveness denotes the proportion of decrease of the confirmed cases due to an intervention. We assume the sensitivity of front door screening of 0.5 or 0.7 and the protection rates possibly becoming 0.3, 0.6, or 0.9 as reinforcing protection device. Abbreviations:—PPE: personal protective equipment; DN: doctors and nurses; PC: patients and caregivers; ADM: admission; OPD: outpatient department; ER: emergency room.

Next, we simulated the model with various interventions. We set the sum of new incidence for 60 days as an outcome measure. “1- effectiveness of an intervention (%)” is defined as the ratio of the outcome with an intervention to one without any intervention, so effectiveness denotes the proportion of decrease of the confirmed cases due to an intervention. Fig 6 shows the effectiveness of all intervention scenarios and the effectiveness of the detection of infectious patients, 80.7%, is the highest among the control measures. Therefore, an examination of any suspected cases is the most important way to prevent an outbreak in the hospital. Wearing universal masks by medical staff and visitors (see PPE DN 0.3 PC 0.3 in Fig 6) shows about 66.4% of effectiveness. The impact of different protection rates of medical staff tested by three scenarios (0.3, 0.6, or 0.9) turned out to be insignificant (66.4%, 67.8%, or 68.9%, respectively). In other words, reinforcement of PPE for medical staff does not show an expected improvement of effectiveness. Quarantine of new hospitalized patients is another effective way to prevent outbreaks. The quarantine unit is as effective as 65.7%, while front door screening shows less effectiveness, which is up to 43.1%. The screening of patients who are admitted to the ward is the most effective method, followed by ER and OPD (30.7%, 28.7%, and 2.2% with sensitivity of screening of 0.5). As expected, the more accurate the screening is, the more effective it is as a control measure. If the sensitivity of screening is 0.7, the effectiveness of front door screening for inward, ER, and OPD is 43.1%, 40.1%, and 3.3%, respectively. According to our mathematical model, screening of an OPD is not a good measure to prevent an outbreak in a hospital setting.

### Sensitivity analysis

**Fig 7:**
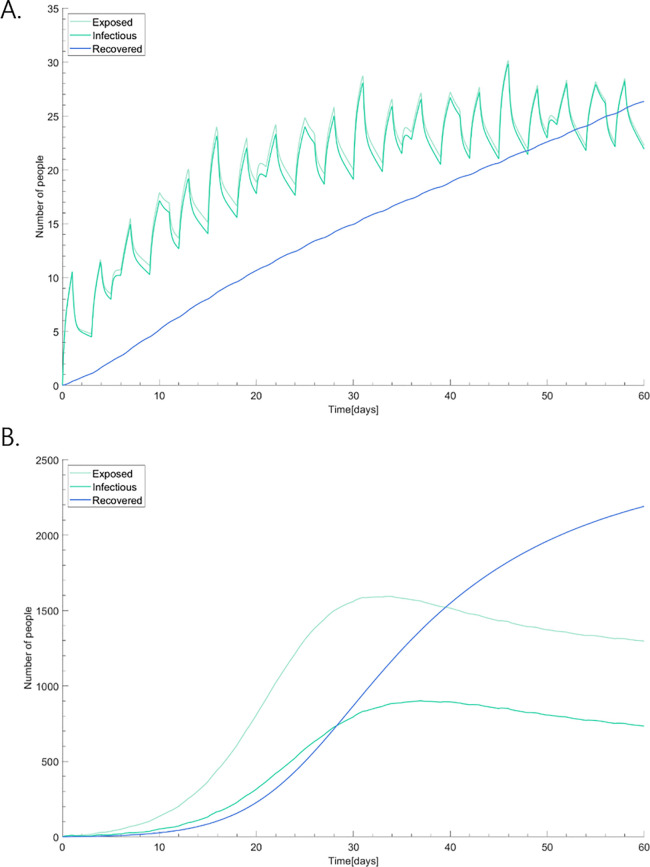
Sensitivity analysis of epidemic curves in the (A) best-case scenario; and, (B) worst-case scenario. The worst-case scenario has a short incubation period and a high reproductive number, resulting in the biggest outbreak in the hospital. On the other hand, the reverse combination yields the best-case scenario.

We performed a sensitivity analysis by taking various combinations of parameter values based on plausible ranges. The worst-case scenario has a short incubation period and high reproductive number, resulting in the biggest outbreak in the hospital. On the other hand, the reverse combination yields the best-case scenario. First, we estimated the outbreak in our hospital in the absence of control measures, which is shown in Fig 7 with sensitivity analysis. The exposed people in the worst-case scenario (Fig 7B) rises to a peak of 902 people, while the exposed people in the best-case scenario (Fig 7A) only reaches 30 people. A highly transmissible case is hard to control with an exponentially increasing number of exposed and infectious patients.

**Fig 8:**
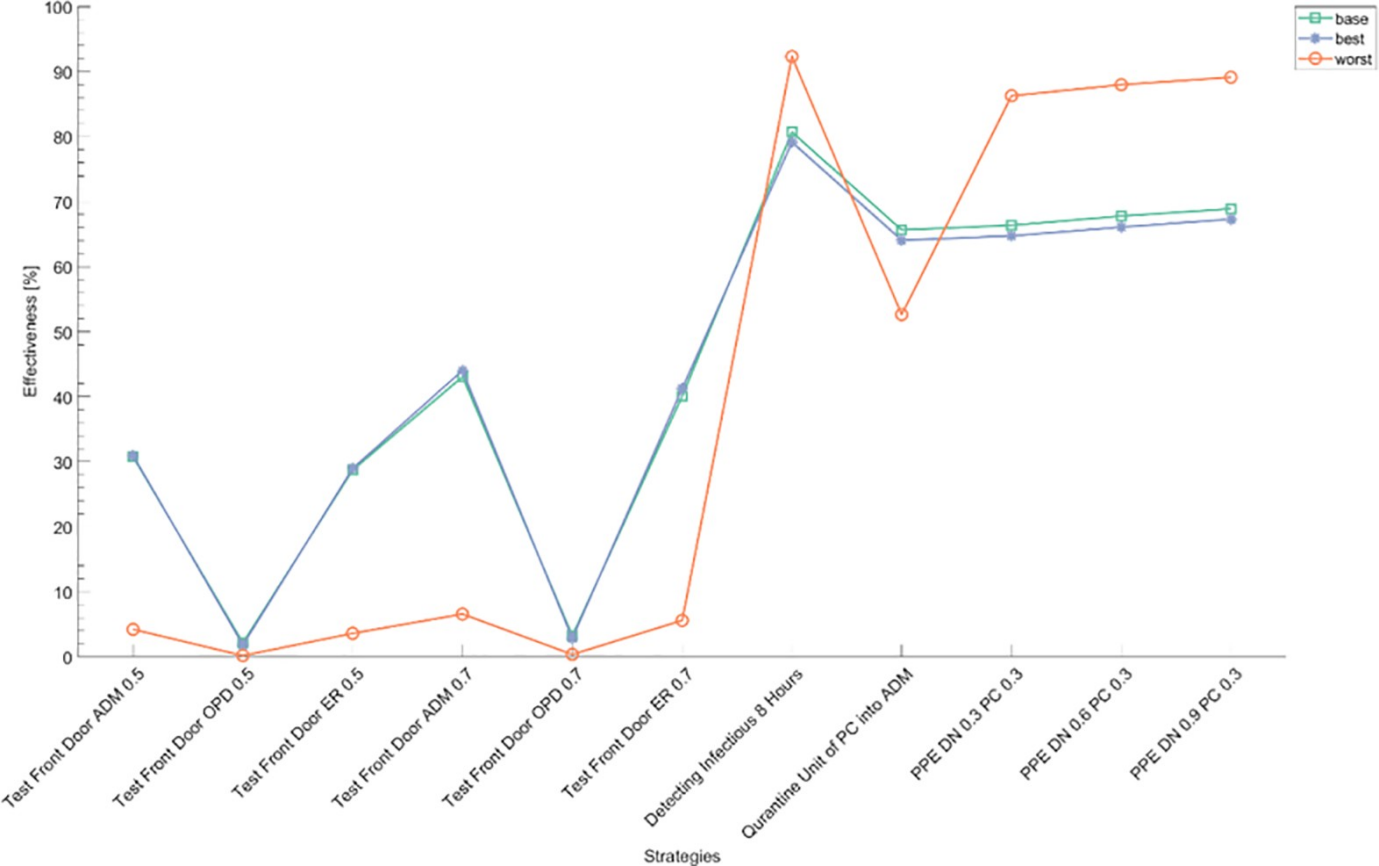
Effectiveness of control measures in three scenarios: Base, best, and worst. Sensitivity analysis shows diagnosis of the disease within eight hours and isolation is still the best intervention in the hospital. The effectiveness of front door screening and the quarantine unit is higher in the best-case scenario, with that of protection device and testing higher in the worst-case scenario. Abbreviations:—PPE: personal protective equipment; DM: doctors and nurses; PC: patients and caregivers; ADM: admission; OPD: outpatient department; ER: emergency room.

Additionally, we evaluated the effectiveness of different interventions in three scenarios: base, best, and worst cases (Fig 8). The effectiveness of control is higher in the best-case scenario through front door screening and use of a quarantine unit. Screening of inpatients through front door screening with a sensitivity of 0.7 shows effectiveness of 44.0% and 6.6% in best- and worst-case scenarios, respectively. Regarding the ER, the effectiveness is 41.3% in the best-case scenario and 5.6% in the worst-case scenario. Effectiveness is about seven times higher in front door screening of admission wards and ERs without reference to sensitivity of screening. In a low transmission setting, it is crucial to detect the patients with COVID-19 from ER and in wards before inflow to a hospital. A quarantine unit also helps prevent the outbreak in the hospital more in the best-case scenario than in the worst (64.1% vs 52.6%). A high transmission rate in the hospital offsets the effort of screening of infectious patients and the short incubation period decreases the efficacy of the quarantine of newly hospitalized patients.

On the other hand, the probability of control by testing and protection devices is higher in worst-case scenarios. PPE reinforcement decreases transmission effectively in highly transmissible conditions, which contributes to reducing the outbreak as with the worst-case scenario. Strengthening protection has little impact on the new incidence of confirmed cases in sensitivity analysis. Testing to detect and isolate confirmed cases is also more important in higher transmission cases (92.3%). Sensitivity analysis shows diagnosis of the disease within eight hours and isolation is still the best intervention strategy in a hospital.

## Discussion

Since the global outbreak of COVID-19, many control measures have been implemented to try and contain the pandemic: isolation of confirmed and suspected cases; contact tracing; social distancing; and, travel restrictions. Suggestion of best strategies which offer greater benefits is difficult in the context of an epidemic. Several mathematical models have been proposed to explain the system and help decision making beyond hospital settings [19–22]. One study explored the spatial association of the early stages of the COVID-19 pandemic in China [23]. A dynamic mathematical model estimated that the growth rate of COVID-19 is about twice that of the SARS and MERS, and the doubling cycle is two to three days without intervention [24]. A stochastic transmission model assessed the potential for transmission in locations outside Wuhan, if cases were introduced. It calculated that once there are at least four independently introduced cases, there is a more than a 50% chance that the infection will establish itself within that population [25].

In this study, we simplified the transmission of COVID-19 in a hospital to construct a mathematical model that would enable us to estimate the outbreak and determine the effectiveness of control measures. We conducted a sensitivity analysis with different assumptions because of the uncertainty of the parameters. Early testing of infected cases and monitoring the quarantine ward for newly hospitalized patients are effective ways to minimize the COVID-19 outbreak within a hospital. Detecting the patients with COVID-19 from the ER and in wards before inflow to a hospital is effective in low transmission settings; PPE is important to control transmissibility in high transmission settings. Our results could expand to many interventions implemented in society. In high transmissible and short latent cases, transmission reduction interventions including wearing universal masks are more important than restriction of inflow of patients by screening and isolating suspected cases. Above all, quarantine and isolation efficacy should be increased by means of proper hygiene and personal protection.

To our knowledge, this study is the first model to estimate the epidemics in a hospital and evaluate the effects of control measures for COVID-19. Other studies about hospital outbreaks from infectious diseases were conducted with many different mathematical models: a multi-agent model or SEIR transmission model [26–29]. In the case of MERS-CoV, the emergency departments exercised great influence over the epidemic size for both patients and healthcare workers, and isolation and related strict measures (added PPE or environmental sanitation) suppressed the epidemics with the help of the SEIR compartmental model [26]. The SEIR compartmental model was similar to our model, which is deterministic, multi-type, and spatial in a hospital setting. Our model set the reproductive number with sensitivity analysis, assumed the regular inflow of COVID-19 patients to a hospital, and compared the control measures.

There are some limitations of this model. Our model does not include people who enter the hospital and do not belong to the four occupations specified (doctors, nurses, patients, and caregivers). Weekends, when most of the medical staff are off duty were not taken into consideration. Various units and situations of the hospital have not been included in this model, such as the intensive care unit, operation room, and confirmed cases of medical staff. While the model assumes the same inflow of exposed and infectious people from the patient and caregiver group, the prevalence of asymptomatic infection has not been clarified yet. We set the rate at which the exposed individuals become infectious at 0.1, which has also not been confirmed. In addition, we assumed that the sensitivity of the front door screening is low (0.5 or 0.7) and detects only infectious persons; however, thorough checklists and use of thermometers might detect more infectious or exposed people. Therefore, it is cautious to assert that front door screening is effortless. More studies should be conducted on the outbreak and control measures from different perspectives. Even though we did not include all the details, this study could improve our insights into epidemiological situations and identify which control measures are most efficacious in hospital settings. Though this study was confined to one hospital, it can be tailored to the requirements of other hospitals, facilitating effective hospital-based management during this rapidly evolving outbreak.
