# Correction: A mathematical model of COVID-19 transmission in a tertiary hospital and assessment of the effects of different intervention strategies

**DOI:** 10.1371/journal.pone.0253685

**Published:** 2021-06-17

**Authors:** Yae Jee Baek, Taeyong Lee, Yunsuk Cho, Jong Hoon Hyun, Moo Hyun Kim, Yujin Sohn, Jung Ho Kim, Jin Young Ahn, Su Jin Jeong, Nam Su Ku, Joon-Sup Yeom, Jeehyun Lee, Jun Yong Choi

In the Incubation period and serial interval subsection of the Methods, there is an error in the second sentence. “Serial interval” should be “infectious period”. A reference is also missing in the third sentence. The corrected subsection is as follows:

## Incubation period and infectious period

The incubation period has not been determined yet and we set it at 5.2 days [13] as a base case and 6.4 days [16] for sensitivity analysis. The infectious period has not been determined and we assumed 9.5 days [17] and 4.6 days for sensitivity, which is 2 times of 2.3 days–that is the difference between 7.5 days serial interval and 5.2 days incubation period [13]. Note that these parameter values were to be fitted with different assumptions for distribution [18]. However, in an average sense, they have few differences with other fitting results and can be used as parameters in our model. With these parameters, we set the base-, worst-, and best-case scenarios and performed the sensitivity analysis with them (See Table 4).

The image for Fig 4 is incorrect and appears as a duplicate of Fig 7. The image for Fig 6 is incorrect and appears as a duplicate of Fig 1. Please see the correct figures here.

**Fig 4:**
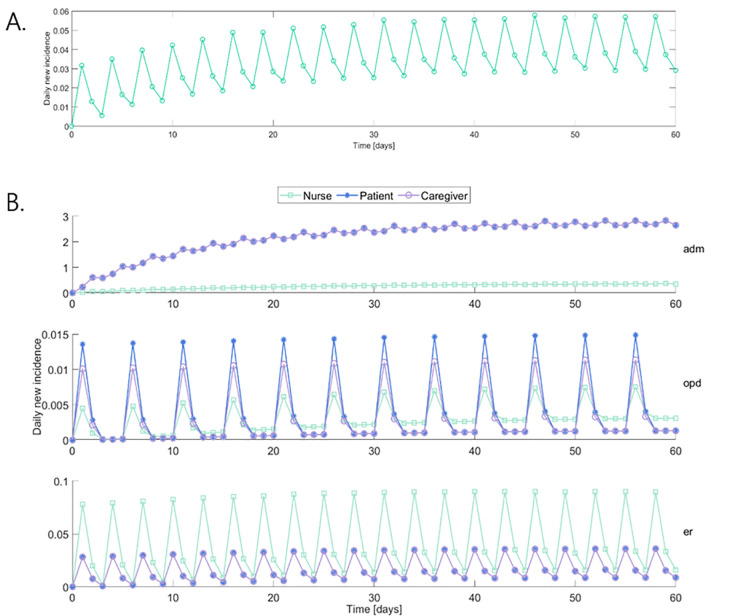
Daily new incidence of COVID-19. (A) Epidemics in doctor status. (B), Epidemics in 10 statuses; from top to bottom, ADM, OPD, ER. Abbreviations:—ADM: admission; OPD: outpatient department; ER: emergency room.

**Fig 6:**
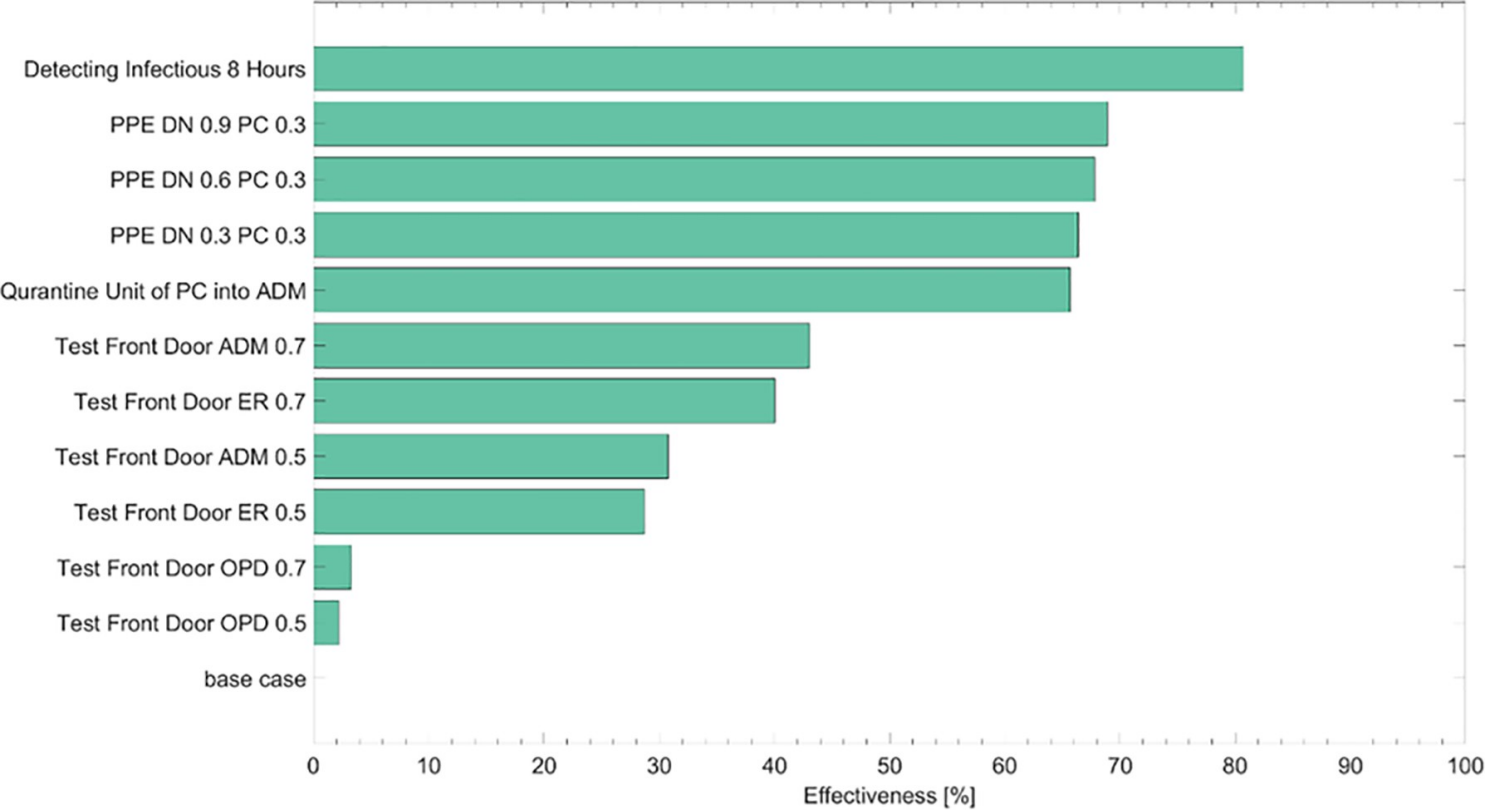
Effectiveness of all intervention scenarios. Effectiveness denotes the proportion of decrease of the confirmed cases due to an intervention. We assume the sensitivity of front door screening of 0.5 or 0.7 and the protection rates possibly becoming 0.3, 0.6, or 0.9 as reinforcing protection device. Abbreviations:—PPE: personal protective equipment; DN: doctors and nurses; PC: patients and caregivers; ADM: admission; OPD: outpatient department; ER: emergency room.

In Table 2, the Symbol and Value of row 4 should be center-justified. Please see the correct Table 2 here.

**Table 2:** The base parameter settings.

Parameter	Symbol	Value
Incubation Period [days]*	1/*f*	5.2
Infectious Period [days]*	1/*γ*	9.5
Impact of the exposed onto the infection×	*ε*	0.1
The average inflow number of ADM from the outside per day†	-	0
The average inflow number of OPD from the outside per day†	-	11242.6
The average inflow number of ER from the outside per day†	-	209.3
The average number from ER to ADM per day†	-	51.4
The average number from OPD to ADM per day†	-	314.6
The rate of outflow from the ADM [1/days]†	-	0.1491
The rate of outflow from the OPD [1/days]†	-	6
The rate of outflow from the ER [1/days]†	-	4

* The incubation period and infectious period are from reference [13], [17]

^×^ Rate at which the exposed persons become infectious

† An average of data collected from the hospital administration department in the study site

Abbreviation:—ADM: admission; OPD: outpatient department; ER: emergency room.

In Table 4, the reference for 1/γ in the Worst Scenario row should be reference 13, not reference 18. Please see the correct Table 4 here.

**Table 4:** Parameter values for evaluation of various interventions and sensitivity analysis^1^.

Scenario	Parameter Set		Source
**Base**	1/*f*^2^	5.2	[13]
	1/*γ*^3^	9.5	[17]
	*R*_0_^4^	2.2	[13]
**Best**	1/*f*	6.4	[16]
	1/*γ*	9.5	[17]
	*R*_0_	2.2	[13]
**Worst**	1/*f*	5.2	[13]
	1/*γ*	4.6	[13]
	*R*_0_	6.47	[14]

^1^ We set the best- and worst-case scenario parameter sets in terms of curbing viral transmission. If the virus has a long infectious period and low reproductive number, the transmissibility is low, which is helpful in curbing the spread of disease. On the other hand, with a short infectious period and high reproductive number, it would lead to high transmissibility even in a restricted condition.

^2^ 1/*f* is the incubation period; a reversal of the rate at which the exposed patients become infectious

^3^ 1/*γ* denotes the infectious period, a reversal of the rate at which the infectious patients would recover,

^4^ *R*_0_ denotes the reproductive number, an average number of secondary cases generated by a case in an entirely susceptible population

The ORCID iDs are missing for multiple authors. Please see the authors’ respective ORCID iDs here:

- Author Yae Jee Baek’s ORCID iD is: 0000-0003-0994-4940 (https://orcid.org/0000-0003-0994-4940).
- Author Yunsuk Cho’s ORCID iD is: 0000-0002-6089-876X (https://orcid.org/0000-0002-6089-876X).
- Author Jong Hoon Hyun’s ORCID iD is: 0000-0002-9621-0250 (https://orcid.org/0000-0002-9621-0250).
- Author Moo Hyun Kim’s ORCID iD is: 0000-0003-3634-0296 (https://orcid.org/0000-0003-3634-0296).
- Author Yujin Sohn’s ORCID iD is: 0000-0001-7018-8641 (https://orcid.org/0000-0001-7018-8641).
- Author Jung Ho Kim’s ORCID iD is: 0000-0002-5033-3482 (https://orcid.org/0000-0002-5033-3482).
- Author Jin Young Ahn’s ORCID iD is: 0000-0002-3740-2826 (https://orcid.org/0000-0002-3740-2826).
- Author Su Jin Jeong’s ORCID iD is: 0000-0003-4025-4542 (https://orcid.org/0000-0003-4025-4542).
